# Ecologo-Geographic Distribution Patterns of the Italian Locust *Calliptamus italicus* (Linnaeus) (Orthoptera: Acrididae) in the Easternmost Part of Its Range

**DOI:** 10.3390/insects16020211

**Published:** 2025-02-14

**Authors:** Michael G. Sergeev, Muratbek K. Childebaev, Rong Ji, Vladimir V. Molodtsov, Natalya S. Baturina, Irina A. Van’kova, Marya N. Kim-Kashmenskaya, Kristina V. Popova, Vasily D. Zharkov, Oxana V. Yefremova

**Affiliations:** 1 Department of General Biology and Ecology, Novosibirsk State University, 1 Pirogova Street, 630090 Novosibirsk, Russia; vv@fen.nsu.ru (V.V.M.); natalya.s.baturina@gmail.com (N.S.B.); mashust@gmail.com (M.N.K.-K.); oxana@fen.nsu.ru (O.V.Y.); 2 Laboratory of Biogeomodelling and Ecoinformatics, Novosibirsk State University, 1 Pirogova Street, 630090 Novosibirsk, Russia; vankova1969@gmail.com (I.A.V.); kristin_belle@mail.ru (K.V.P.);; 3 Laboratory of Invertebrate Ecology, Institute of Systematics and Ecology of Animals, Siberian Branch, Russian Academy of Sciences, 11 Frunze Street, 630091 Novosibirsk, Russia; 4 Institute of Zoology, 93 Al-Farabi Avenue, 050060 Almaty, Kazakhstan; childebaev@mail.ru; 5 Xinjiang Laboratory of Biology for the Protection and Regulation of Species in Special Environment, International Center for the Collaborative Management of Cross-Border Pests in Central Asia, College of Life Science, Xinjiang Normal University, Urumqi 830054, China; jirong@xjnu.edu.cn

**Keywords:** climate changes, range, dispersal, modeling, Maxent, Inner Asia, dynamics, population, plant protection, outbreak risks

## Abstract

Grasshoppers (Acridoidea) are one of the most essential components of grassland ecosystems. They are mainly primary consumers and some of them, e.g., the Italian locust, may be very important pests. The main goal of this article is to discuss possible shifts in the species distribution relative to global and local changes. General patterns of latitudinal, longitudinal, and altitudinal distribution of its populations are revealed for the eastern part of its range (mainly from 70° E eastward). Data on its historic and modern localities with geographic coordinates were collected and analyzed. The Maxent approach was used to model species distribution. The comparative analysis shows there are no significant changes in its range. The analysis of altitudinal distribution over mountains of the region demonstrates no significant changes as well. The expected distribution of *Calliptamus italicus* based on the location points displays significant changes in areas suitable for the species and their evident associations with outbreak occurrences. The models created for 2021–2040 and 2041–2060 unveil that, in the future, the local parts of the Italian locust range may shift northward and north-eastward, but the main areas with suitable conditions for this species will remain almost the same.

## Simple Summary

At the end of the 20th century, the Italian locust became one of the most important pests in extratropical Eurasia. However, our knowledge about its ecology and distribution is primarily restricted by species upsurges. This is why opportunities to predict long-term changes in the Italian locust distribution and population dynamics are very limited. In this article, we analyzed the data on the species distribution in the easternmost part of its range for three periods (before 1961, 1961–1997, and 1998–2022) and tried to reveal possible shifts of both the species range boundaries and areas with optimal conditions relative to global and local changes.

## 1. Introduction

Grasshoppers (Acridoidea) are some of the most prominent components of grassland ecosystems, such as steppes, prairies, semi-deserts, and savannas [1]. They are mainly primary consumers in the local food chains and their activity commonly accelerates fluxes of energy and substances in the ecosystems. However, from time to time, some acridid species can reproduce en masse, and huge outbreaks may start. As a result, these insects are able to destroy all or almost all aboveground parts of herbaceous plants in the natural grasslands and they can seriously damage agricultural and hay fields and pasturelands.

At the end of the last century, the Italian locust, *Calliptamus italicus* (Linnaeus), became one of the main acridid pest species in Eurasia. During the 20th century, its major outbreaks were mainly confined to three particular decades: 1921–1930, 1931–1940, and especially 1991–2000. In 2000, in Kazakhstan and Russia, more than 16,600,000 ha were plagued, and various insecticides, from more organophosphates to IGR (insect growth regulators), were used across the area above 10,000,000 ha [1]. In 2000, Kazakhstan spent more than USD 23,000,000 for control operations [1].

This locust is a common and often abundant acridid over the dry steppes, semi-deserts, northern deserts, and the dry Mediterranean landscapes, from south Europe to the Altai Mts. and from Middle Russia to Turkey, Turkmenistan, and the northwest of China [2,3,4,5,6,7,8,9,10]. Some traits distinguish it from typical gregarious acridid species (i.e., typical locusts) [4,11]. The coloration patterns of both gregarious and solitarious forms are the same, but the gregarious specimens have relatively long wings [4,11,12]. Its gregarious adults can fly actively and demonstrate explicit migrations over comparatively small distances, usually about 100–200 km [1,13,14]. The hopper bands can migrate as well but commonly stay in almost the same place for several weeks if there is enough food [3,15]. Sir B. P. Uvarov [10] noted that our knowledge regarding the bionomics and population ecology of the species, especially betwixt its upsurges, is insufficient. Over the last three decades, more than several dozen studies concerning this species have been issued, but almost all have been confined to plague periods [1,2,3,4,14,15,16,17,18].

An analysis of the spatial population distribution of *C. italicus* between outbreaks reveals various habitat preferences in the different parts of its range. In the northern (forest-steppe) part, it colonizes very dry habitats, in the central one (steppes and semi-deserts), its local populations are common across comparatively xeric and mosaic ecosystems, and in the southern part, it is normally located either in meadows of flood plains or in the mountain steppes [2,3,4,19]. The species likes very heterogeneous semi-arid landscapes (particularly with sagebrushes *Artemisia* spp.) associated with the dry steppes, semi-deserts, and the Mediterranean region as well. Throughout upsurges, the Italian locust may pierce and occupy different agricultural fields, both cultivated and abandoned, but because the species mainly prefer to feed off dicotyledons, it may primarily consume some weeds on fields of cereal grain crops.

The easternmost part of the Italian locust range (from the south of West Siberia to the Dzhungarian (Jungarian) Basin in Northwestern China (Xinjiang) and the Pamiro-Alay Mts. in Kyrgyzstan, Tajikistan, and Uzbekistan) is one of the main areas of its plagues. Its numerous and serious upsurges were observed here during the 20th century. Now, this area is deserving of more attention, especially relative to the modern global and local transformation of ecosystems. In this context, ecologo-geographic modeling can help us to reveal some main trends in the Italian locust distribution changes.

First, cartographic models of the Italian locust spatial distribution across the eastern European and Asian parts of its range were developed in the 1920s–1930s. I. N. Filipjev [20] depicted the wide band of possible species outbreaks crossing the grassland areas from the Nistru (Dniester) River Basin to the Kulunda steppe [4]. Slightly later, the regionalization scheme based on the pest species distribution was suggested for the territory of the former USSR [21]. The authors included the areas of possible outbreaks of the Italian locust, not only the steppes of Kazakhstan and West Siberia but also the semi-deserts of the region from the Volga River Basin to the Altai Mts. [4].

In 1962, K. A. Vasil’ev [13] described the main region of the Italian locust outbreaks in the semi-deserts of Kazakhstan (especially with sandy soils) and depicted some additional terrains of possible upsurges in the sandy steppes near the Irtysh River. Later, O. F. Fedosimov and N. G. Telepa [22] summarized data on infested and protected areas and showed that the dry steppes and the semi-deserts of Kazakhstan were key territories of the Italian locust harmfulness. However, they noted the evident association of the upsurges with climatic fluctuations. In any case, all printed maps of the 1920s–1980s show quite similar patterns of the Italian locust outbreaks’ distribution, especially their association with the dry grasslands.

In the 1970s, another approach was used to analyze the Italian locust distribution across southwest Siberia and Kazakhstan [23]. Its local population distribution over the system of regional and local transects crossing life zones and local ecosystems was described based on the species abundance data. These studies allowed more complicated patterns to be revealed concerning species distribution during periods between upsurges. Two optimal areas, i.e., terrains where the species local populations occur in all or almost all applicable habitats, were described for the Italian locust: the dry parts of the steppes between the Irtysh and Ob Rivers and the semi-deserts of the Saryarka (Kazakh Uplands) [23]. Later, this approach was developed. The data from several long transects crossing the species range in its several parts were combined, and the species distribution was mapped on this basis [24,25,26,27]. The first variant of the manually drawn map of the species population distribution across the eastern part of its range during periods between outbreaks was published in 1997 [2]. The revealed distribution pattern confirmed early published results [13,20,21,22,23], e.g., the species explicitly prefers the semi-deserts of Kazakhstan. In addition, there is another optimum in the typical southern steppes. This optimum also includes the local dry pine forests. Outbreaks of the Italian locust were and are very common here [3,4,18]. However, in West Siberia, the Italian locust is actually distributed more widely across the local southern forest-steppes and northern steppes than was previously indicated by [23]. Later, this map was slightly modified [4,26]. The presence of two optima in the easternmost parts of the species range evidently coincides with the pattern revealed by M. V. Stolyarov [28] for West Kazakhstan. Both optima look like two stripes stretching from west to east through the Asian part of the Italian Locust range. Both stripes are characterized by complicated long-term dynamics and irregular outbreaks of the Italian locust populations [3,4,18,29]. However, in the semi-deserts, *C. italicus* remains the common species between upsurges. On the contrary, in the steppes, its abundance can be extremely low for many years, but the species’ local populations exist constantly in natural and semi-natural grasslands [3]. Consequently, the locust outbreaks may be unexpected here [3,22].

Qin Yujia and co-authors [30] tried to use the CLIMEX model (based on some estimations of species tolerance) to forecast the possible distribution of the Italian locust in China and showed that according the model, the optimal climate conditions for the species are in the northern parts of China (north of the Yangtze River). The authors also estimated some possible shifts in the Italian locust distribution for the middle of the 21st century and showed very low levels of these changes. However, ecological parameters of the Italian locust were almost arbitrarily selected from different sources and for quite different species populations. For instance, the authors suggested that the diapause period may continue for 90–120 days, but it can be more than 210 days in the northern parts of the Italian locust range [1,4]. Moreover, the main portion of the Italian locust range is inside the area influenced by the mid-latitude spring/summer westerlies and with main rainfalls during spring and the beginning of summer [4,5]. On the contrary, the range of *C. abbreviatus* Ikonn. occupies mainly the territory with rainfalls in the middle and end of summer (areas with dominance of intracontinental air masses or summer monsoons) [3,9]. Furthermore, the roles of different ecological factors for local populations of *C. italicus* may be quite diverse [3,4,18,31]. In addition, the authors noted that the Italian locust was introduced in Xinjiang in 1999; however, the species occurred in the region at least in the 1950s [32,33].

The aims of our researches are (1) to debate some issues emerging from long-term investigations of distribution patterns of the Italian locust populations across the eastern portion of its range (from 70° E eastward) where huge upsurges of this species occurred during last three decades and (2) to create ecologo-geographic models of the species distribution through Inner Asia as the framework to reveal some possible changes in the future.

## 2. Materials and Methods

### 2.1. Study Territory

Primary data were compiled from 1976 until 2022 in the southern part of the West Siberian Plain, in the eastern and south-eastern parts of Kazakhstan, Kyrgyzstan, Uzbekistan, Tajikistan, and in NW China (Xinjiang). The Altai–Sayan Mts. mainly borders this area on the east. The southern border of the taiga life zone (about 56° N) is normally determined as the northern boundary of the territory, and the subtropical deserts (about 37° N) are defined as the southern one. Until the beginning of the 20th century, this huge region was mainly covered by forests, grasslands, and deserts. During the last hundred years, the main part of local ecosystems was mainly replaced by agricultural lands (fields and pastures) [34,35].

In the region studied, the forest-steppe life zone was originally between 54° N and 55.5° N. The steppes are distributed amid 50.5° N and 54° N. The semi-deserts occupy territories between 47.5° N and 50.5° N, and the deserts are mainly distributed over the local plains and hills further south than 47.5° N. Moreover, there are some mountain systems; among them are the Altai-Sayan Mts., Tarbagatai, Tien (Tian) Shan, Pamiro-Alay, and the Pamirs, with their well-developed altitudinal belts from the dry piedmont plains up to either the nival or alpine levels. Meadows, forest patches, sandy areas, solonchaks (with saline soils), and swamps are also on floodplains along local rivers and around lakes. Across the local plains, mean temperatures are relatively modest (average temperatures of the warmest month are from 17 °C to 31 °C, the same for the coldest month—from 2 °C to −20 °C), and annual precipitation varies amongst 125 and 520 mm [35].

### 2.2. Field Studies

Quantitative and qualitative samples collected in natural, semi-natural, and transformed ecosystems, usually in July and August when adults dominated, were used to reveal the species distribution patterns [11,36]. Depending on the circumstances, three different quantitative methods were used. First, samples captured during a fixed period of time were performed in each habitat studied [2,37,38]. In this case, individuals were collected with a standard entomological net (40 cm diameter) for 10–30 min. The results for each habitat were recalculated to an hour. Second, the standard sweep nettings were performed (sweep numbers commonly varied between 50 and 200) [2,37]. The results were converted to 100 sweeps. Third, we evaluated acridid densities on randomly distributed plots 0.25 × 0.25 m^2^ (sometimes—0.5 × 0.5 m^2^ or 1 × 1 m^2^) [2,37]. Two or three methods were frequently used simultaneously. In addition, we also separately collected specimens to reveal local species diversity. After 1998, we used the Glonass/GPS receivers (Garmin International Inc., Olathe, KS, USA and Magellan (MiTAC Digital Corp.), Newark, CA, USA) to evaluate the geographic positions of localities. We exploited Google Earth Pro (©Google, 2022) to find the geographic coordinates for points studied before 2000. Almost all specimens are in the collections of Novosibirsk State University, the Institute of Systematics and Ecology of Animals (Novosibirsk), and the Institute of Zoology (Almaty). At the beginning of June 2021 and 2024, we arranged special field trips to obtain some additional data concerning the Italian locust distribution across the steppes of the South Ural and the Kulunda steppe (SE West Siberian Plain) accordingly to validate the models.

### 2.3. General Distribution of the Italian Locust in Inner Asia and the Identification Problems

The Italian locust is not known from the mountains of south Siberia (except their north-western, western, and south-western surroundings) over almost all of Mongolia and the Transbaikal region (Dauria). All data on its distribution over this territory [30,39,40,41,42] look like they are based on some misidentifications of their relatives: either *C. barbarus* (Costa) or *C. abbreviatus* (Ikonnikov) [1,4,5,6,7,8,9,43,44,45,46,47,48]. Unfortunately, N. Jago [40] mentioned no specimens of the Italian locust from south Siberia and Mongolia and cited no publications related to the species distribution across this area. As a result, an issue arises as to why he included south Siberia (up to the Amur River Basin) and north Mongolia in the species range. Morphology and coloration patterns of the species of the genus *Calliptamus* Audinet Serville may vary significantly between and inside their populations [4,40,43,44,48]; this is why, in many cases, we can not use only a trait or a few characteristics to distinguish the species or subspecies [4,48]. For instance, orthopterists often use coloration of hind winds to differ *C. italicus* and *C. abbreviatus*, but in some populations, specimens of *C. abbreviatus* with pink hind wings were found [49], and, on the contrary, hind wings of some adults of the Italian locusts may be almost colorless [4]. The main differences between these similar species are characterized in Table 1. The analysis of other published data (either pictures or morphometry) on the species distribution in south Siberia and Mongolia [39,41,42] shows that *C. abbreviatus* occurs across these territories.

### 2.4. Data Analysis

Besides our field data, we also analyzed some old findings, especially compiled by the expeditions of Novosibirsk State University (1961–1981), the Institute of Systematics and Ecology of Animals (the former Biological Institute, Novosibirsk, Russia), the Institute of Zoology (Almaty, Kazakhstan), and Xinjiang Normal University (Urumqi, China). The methods described in Section 2.2 were usually applied. We also used data from various published sources [13,32,50,51,52,53,54,55,56,57,58,59,60,61,62,63,64,65] and data from the museums of the Zoological Institute (Saint Petersburg, Russia), Novosibirsk State University, and the Institute of Systematics and Ecology of Animals. The dataset was divided into three subsets: (1) before 1961 (because in the 1960s, more or less systematic studies of Orthoptera were started in this huge area; often, they included quantitative sampling) (96 localities); (2) from 1961 until 1997 (in 1998, the last huge outbreak of the Italian locust began) (104), and (3) from 1998 until 2022 (178) (see Supplementary Table S1). We also used our new data (5 localities, 2021 and 2024, see Section 2.2) and the data from the Global Biodiversity Information Facility [39] for the region (28 localities, observations for 2012–2024) to validate our models, but, in this case, we did not consider several locations associated with some problematic identification of the species.

The known geographic coordinates and a Lambert conformal conic projection were used to produce the species distribution maps with QGIS 3.18.3. We harnessed the Maxent 3.4.4 software [66,67,68,69] to tailor the species distribution over the region for four sets of data (before 1961; 1961–1997; 1998–2022; all data until 2022). We selected this software since it is relatively formalized [68] and its interface is relatively friendly.

To model the species distribution, we employed the data of WorldClim 2 [70,71], namely the “Historical climate data” (19 standard annually averaged bioclimatic variables at the 30 arcsecond spatial resolution) and “Future climate data” (19 standard averaged bioclimatic variables) for 2021–2040 and 2041–2060 downscaled from two global climate models [71], particularly CNRM-ESM2-1 (Centre National de Recherches Météorologiques and Centre Européen de Recherche et de Formation Avancée en Calcul Scientifique, France) [72,73] and GISS-E2-1-G (NASA Goddard Institute for Space Studies, USA) [74,75] at the 30 arcsecond spatial resolution and for the three Shared Socioeconomic Pathways (1-2.6, 2-4.5, 3-7.0) [76].

Undoubtedly, this approach has some limitations. The Maxent models are restricted by the presence data based on the number of occurrences, chosen parameters of modeling, and selected sets of variables [66,67,68]. The WorldClim dataset contains spatially interpolated data on climates and elevations, and their actual reliability is partly determined by densities of weather stations (relatively low for the region studied) [70]. In any case, we tried to explore the maximum sets of relevant bioclimatic variables to equate results for the same territory but for diverse periods and climatic models. The AUC (the area under the receiver operating characteristic curve) value for sets of 25 replicates with cross-validation was used to estimate accuracy. The significances of climatic variables models were estimated by their predictive contributions and Jackknife tests. We generated the models with the following parameters: features—auto, output format—cloglog [66], and regularization multiplier = 1.

The upper altitudinal limits were compared by the Spearman rank-order correlation coefficient as well.

## 3. Results

### 3.1. Latitudinal and Longitudinal Distribution of the Italian Locust over the Region

**Figure 1:**
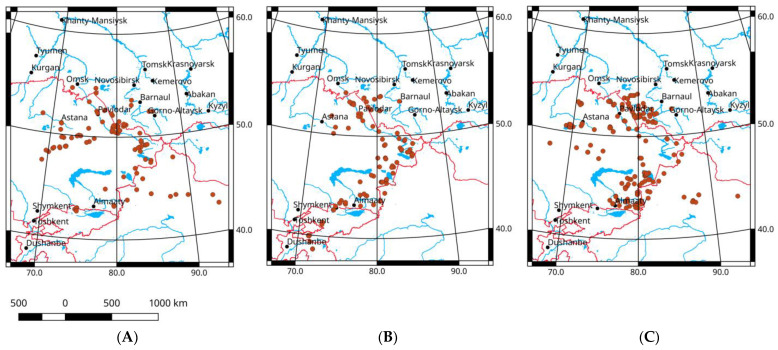
Distribution of *Calliptamus italicus* over the easternmost parts of its range: (**A**)—before 1961; (**B**)—from 1961 until 1997; (**C**)—from 1998 until 2022.

In the eastern part of the range, the Italian locust is bordered by the latitude of 55° N on the north, the Altai-Sayan Mts. (including Salair Range) on the east, the western parts of Gansu and the central parts of Qinghai on the south-east, and the northern boundaries of the Taklamakan Desert on the south (Figure 1). However, some populations of the species are in the highlands of Tien Shan, Pamiro-Alay, the Pamirs, and Hindu Kush. Sometimes, swarms of the Italian locust are able to cross its range and borders. In such cases, they can cross the latitude of 55° N. Several populations were also found on the right (eastern) side of the Ob River valley but the status of these populations is unknown. Some of them may have been eliminated during the last few decades.

The comparative analysis of the model species distribution across the region studies for three periods, before 1961, from 1961 until 1997, and from 1998 until 2022 (Figure 1), shows very similar patterns. No significant disagreements amongst them are revealed and no evident range shifts are observed. Some noticeable distinctions of the distribution maps are determined by different levels of field studies. For instance, we have very limited data concerning the orthopteran distribution in Xinjiang in the 1960–1970s.

The general pattern of the Italian locust distribution over different habitats was previously described for the region [2,3,4,19]. In its northern part, the Italian locust inhabits the southern forest-steppes but usually very locally. It occurs mainly in the overgrazed steppe pasturelands on the low level of abundance. In the steppes, its populations are distributed across both the local drained plains and the dry parts of lower terraces and upper flood plains. In addition, the species colonizes the stony southern slopes of hills as well. In the region studied, the local optimum of the Italian locust is revealed in the dry steppes of Kulunda [2]. Huge outbreaks may develop here [1,2,3,4]. To the south, the Italian locust evidently favors the semi-desert of the eastern part of Kazakhstan. Nearly always, high densities and a presence over all of the available ecosystem types suggests that this territory is optimal for the species [1,2,3,4]. In the arid areas of Kazakhstan and Xinjiang, once can find very local but dense colonies of *C. italicus*. They are often distributed across plains and foothills. Their local density could sometimes reach more than 100 ind./m^2^ [77]. In addition, numerous colonies of the Italian locust often occur in desert river valleys and lake basins. Of note, this species is common across irrigated fields, including alfalfa, and canal sides. Thus, the Italian locust distribution in the easternmost parts of its range does not exhibit explicit consequences of global warming or significant transformations of local ecosystems (especially during the so-called Virgin Land campaign in the 1950s).

### 3.2. Altitudinal Distribution

The Italian locust is not known from the northern part of the Altai Mts. (Cherginskij, Anuiskij, and Bastshelakskij Ridges) (Figure 1). Its scarce colonies are distributed only over the local steppe piedmont plains. In the north-western part of the Altai Mts., the species inhabits the steppe altitudinal belt (Table 2).

The upper altitudinal limit of the Italian locust is 600 m in the north-western part of the Altai Mts. (51°20′ N) and increases as one moves south to reach 2900 m in the Pamirs (38°40′ N). The species prefers the altitudinal belts with dominance of dry grasslands. However, in the northern part of Tien Shan (Dzhungarian Alatau), there are some scarce populations near the timber-line, on the dry alpine meadows, and across the mountain xerophytic vegetation. Such populations were found in the 1960s and described in the 1970s [59]. They look more or less isolated from the main populations distributed below at 1300–1500 m. Global warming may result in increasing their number and abundance.

The upper altitudinal limit of the Italian locust correlates negatively with the latitude (Rs = −0.989, *p* = 0.00000001—without mentioned populations near the timber-line). If one removes data for the 21st century, the correlation remains almost the same (Rs = −0.979, *p* = 0.000066—also without populations near the timber-line). This means that there were no significant changes in the Italian locust altitudinal distribution during the last decades in the easternmost parts of its range.

### 3.3. Ecological Models of the Species Distribution

**Figure 2:**
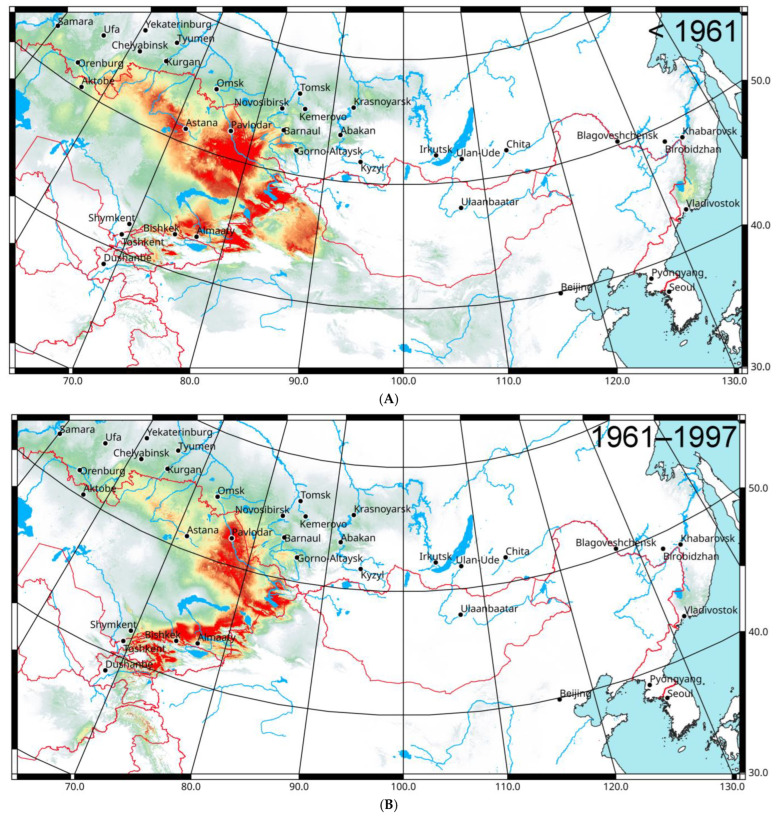

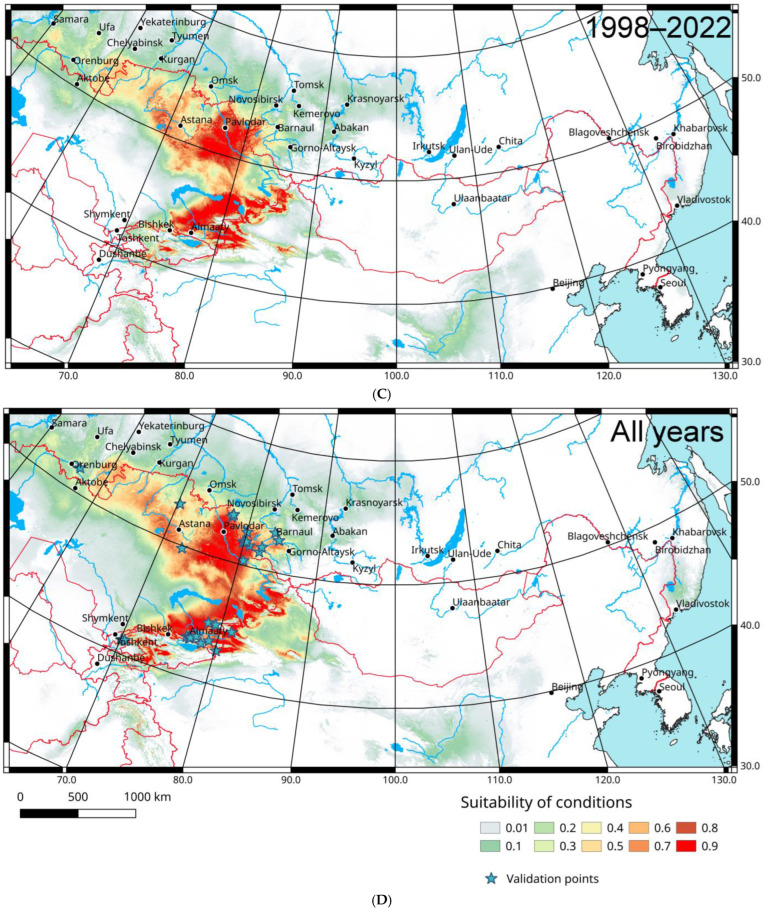
Predicted probabilities of suitable conditions for *Calliptamus italicus* in the eastern part of its range (bioclimatic variables for 1970–2000; point-wise means for 25 replicates): (**A**)—distribution data before 1961; (**B**)—distribution data (1961–1997); (**C**)—distribution data (1998–2022); (**D**)—all distribution data and validation localities. Asterisks—validation localities (see Section 2.2 and Section 2.3).

The predicted distribution patterns relative to climatic data for 1970–2000 were analyzed and compared for three different periods: before 1961, from 1961 until 1997, and from 1998 until 2022 (Figure 2). The backward forecast based on the localities identified before 1961 shows that the optimal environment for the species was mainly in the eastern part of Kazakhstan (except the local deserts) and in the dry and typical grasslands of the south-eastern part of West Siberian Plain (between Pavlodar and Barnaul) (Figure 2A). Some terrains with relatively suitable conditions are in the dry steppes of north-west Kazakhstan, the southern part of the Chelyabinsk Region, and in the northern part of Xinjiang. The pattern corresponds to data on the distribution of the species unpsurges in the dry steppes and semi-deserts of central and east Kazakhstan in the 1940s [13].

**Figure 3:**
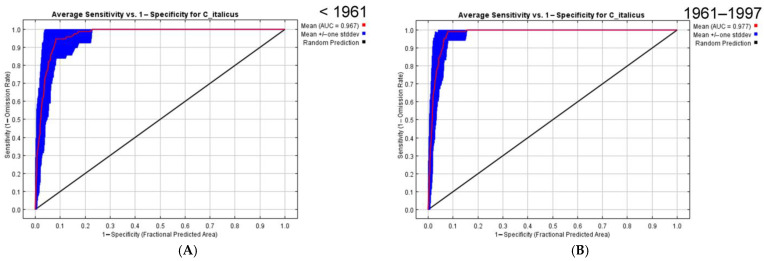

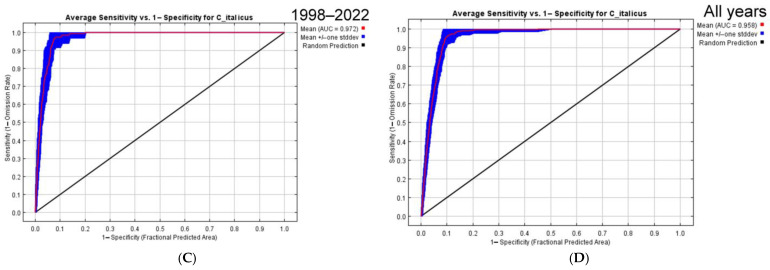
Reliability tests for the *Calliptamus italicus* distribution models in the eastern part of its range (bioclimatic variables for 1970–2000; 25 replicates with cross-validation): (**A**)—distribution data before 1961; (**B**)—distribution data from 1961 until 1997; (**C**)—distribution data from 1998 until 2022; (**D**)—all distribution data.

**Figure 4:**
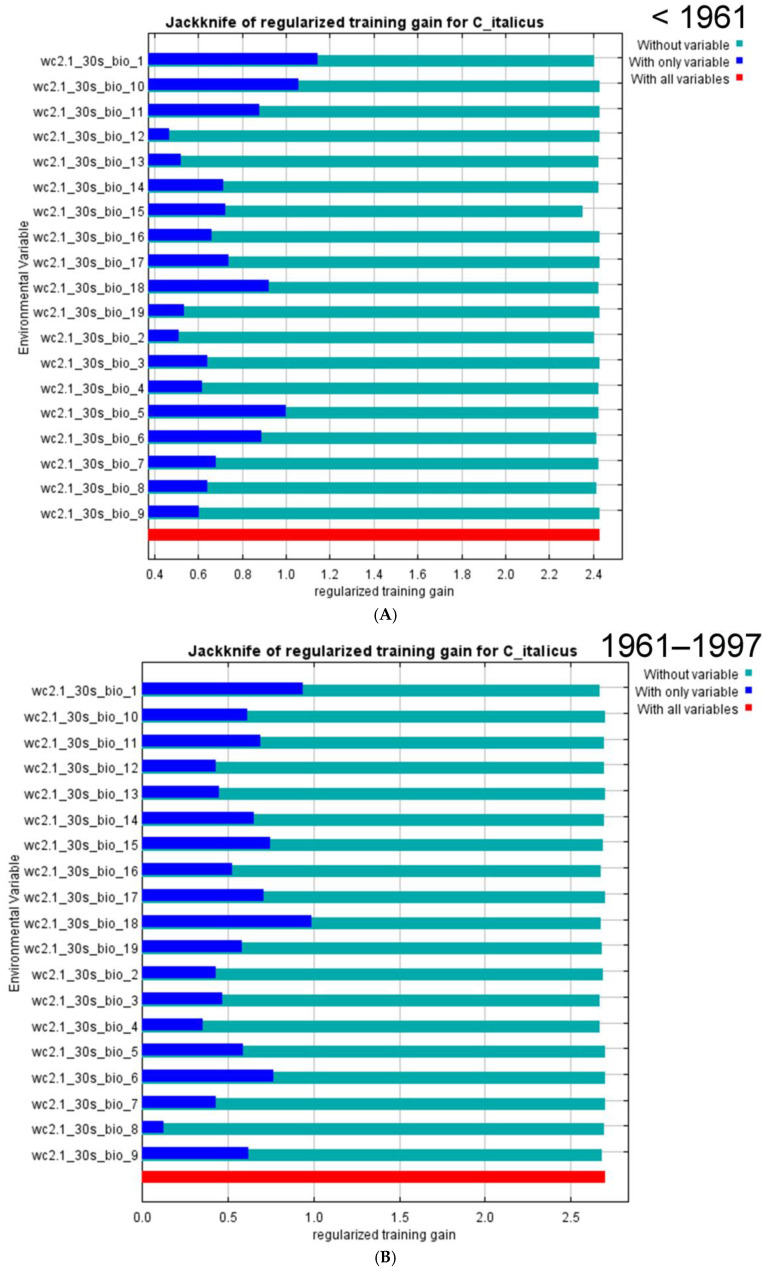

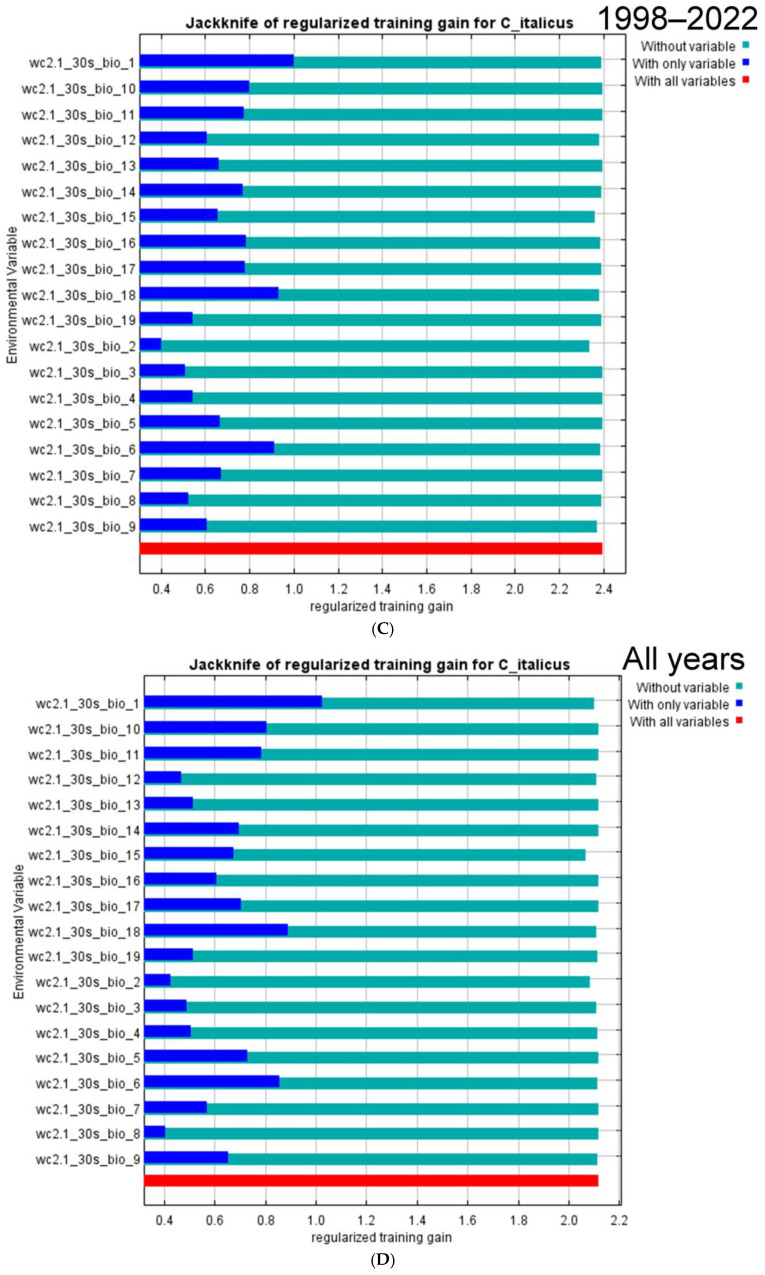
Jackknife of the regularized training gain for the *Calliptamus italicus* distribution models in the eastern part of its range (bioclimatic variables for 1970–2000; 25 replicates with cross-validation): (**A**)—distribution data before 1961; (**B**)—distribution data from 1961 until 1997; (**C**)—distribution data from 1998 until 2022; (**D**)—all distribution data.

For the period before 1961, the area under the receiver operating characteristic curve (AUC) value is 0.967 (Figure 3A). This means the model is well supported. Several climatic variables, namely annual mean temperatures (bio_1), mean diurnal ranges of temperatures (bio_2), and variation in seasonal precipitation (bio_15), are the most important (Table 3). Two other parameters (the mean temperature of the warmest quarter (bio_10) and the maximum temperatures of the warmest month (bio_5) can be added on the basis of the the Jackknife test results (Figure 4A). All variables are important for the growth of nymphs and adults.

The forecasted distribution of *C. italicus* predicated on the occurrence data between 1961 and 1997 referring to climatic data for 1970–2000 (Figure 2B) looks slightly different. Many areas in north and east Kazakhstan, in south Siberia, and NW China became less suitable for the species. Really, at that time, its outbreaks were rare and weak [3,4]. Such shifts might be determined by the so-called Virgin Land campaign in the 1950s that resulted in the deterioration of many landscapes including those that were fitted earlier for the Italian locust. However, some parts of Tien Shan look like very suitable for the species. This pattern correlates with the very strong and unusual outbreak of *C. italicus* in the central parts of these mountains area in 1976–1981 [79].

For 1961–1997, the model performance is high as well with an AUC value 0.977 (Figure 3B). The main factors are mean diurnal ranges of temperatures (bio_2), precipitation of the driest quarter (bio_17), and minimal temperatures of the coldest month (bio_6) (Table 3). Two variables (annual mean temperatures (bio_1) and precipitation of warmest quarter (bio_18)) can be added from the Jackknife test results (Figure 4B). That means, in some seasons, that low temperatures of winters in conjunction with thin snow cover can result in a loss of eggs in eggpods.

The foretold distribution of *C. italicus* based on the presence data after 1998 relative to climatic characteristics for 1970–2000 (Figure 2C) displays a significant increase in areas suitable for the species, especially in NE and SE Kazakhstan and in some parts of Tien Shan and the Dzhungarian Basin. Some terrains of the forest-steppes and the south taiga across south Siberia and of the Ordos Plateau in China look like places for possible north- and eastward dispersal of the Italian locust in the future. The model performance is also high (Figure 3C). Mean diurnal ranges of temperatures (bio_2), minimal temperatures of the coldest month (bio_6), annual mean temperatures (bio_1), variation in seasonal precipitation (bio_15), and precipitation of the warmest quarter (bio_18) are the most important variables (Table 3; Figure 4C). This pattern relates to the distribution of the main outbreaks of this species in the eastern parts of Kazakhstan and in the south-eastern parts of West Siberia in 1999–2001 [3,4].

The model for all distribution data is similar (Figure 2D). The areas applicable for the Italian locust cover almost all steppes and semi-deserts between the Ural Mts. on the west and the Altai-Sayan Mts. on the east. Moreover, the northern deserts of Kazakhstan and some parts of Xinjiang and Kyrgyzstan are characterized by very suitable conditions for this species as well. Nearly all these territories may be defined as areas where the Italian locust upsurges can start. In addition to the territories of the forest-steppes and the south taiga in south Siberia, some regions where the species may exist are in the northern mountain parts of Pakistan, over the Ordos Plateau (China), and in the southern part of the Russian Far East. The most significant variables for all data are annual mean temperatures (bio_1), mean diurnal ranges of temperatures (bio_2), variation in seasonal precipitation (bio_15) (Table 3), precipitation of the warmest quarter (bio_18), and minimal temperature of the coldest month (bio_6) (Figure 4D).

The distribution of many applicable validation localities (2012–2024) [39] (see also Section 2.2 and Section 2.4) matches the high values (commonly > 0.5) of the predicted probabilities of suitable conditions (from 0.08 to 0.91 with mean ± s.e. = 0.48 ± 0.04 and median = 0.47).

**Figure 5:**
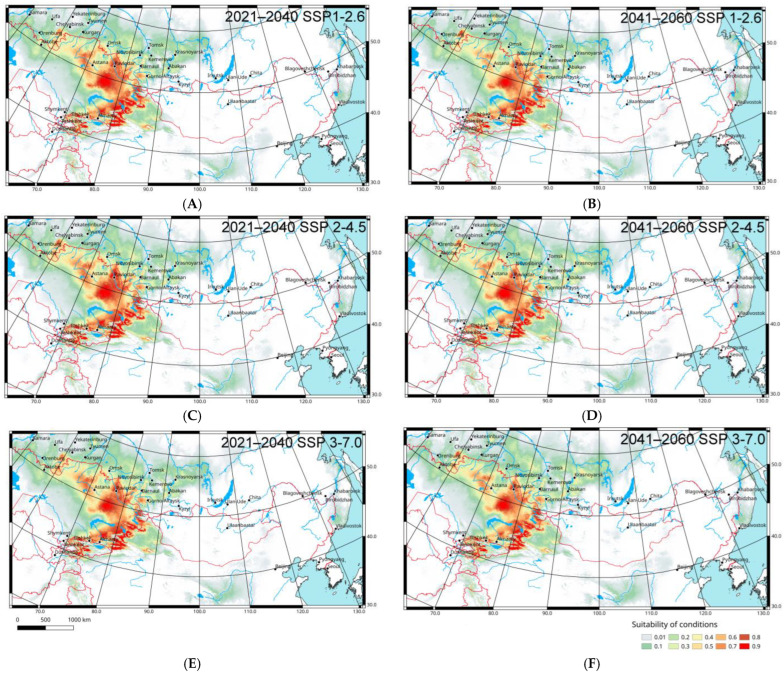
Predicted probabilities of suitable conditions for *Calliptamus italicus* in the eastern part of its range (all distribution data; forecasts of bioclimatic variables for 2021–2040 and 2041–2060 according the global climate model CNRM-ESM2-1 [72,73]; point-wise mean for 25 replicates): (**A**,**C**,**E**)—2021–2040; (**B**,**D**,**F**)—2041–2060; (**A**,**B**)—the 1-2.6 Shared Socioeconomic Pathway based on low greenhouse gas emissions; (**C**,**D**)—the 2-4.5 Shared Socioeconomic Pathway based on intermediate greenhouse gas emissions; (**E**,**F**)—the 3-7.0 Shared Socioeconomic Pathway based on high greenhouse gas emissions [76].

**Figure 6:**
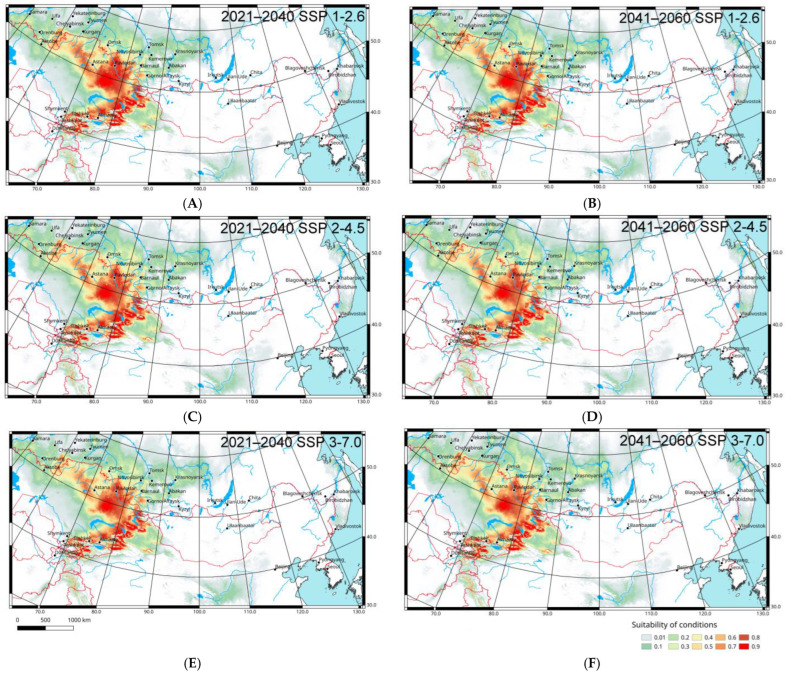
Predicted probabilities of suitable conditions for *Calliptamus italicus* in the eastern part of its range (all distribution data; forecasts of bioclimatic variables for 2021–2040 and 2041–2060 according the global climate model GISS-E2-1-G [74,75]; point-wise mean for 25 replicates): (**A**,**C**,**E**)—2021–2040; (**B**,**D**,**F**)—2041–2060; (**A**,**B**)—the 1-2.6 Shared Socioeconomic Pathway based on low greenhouse gas emissions; (**C**,**D**)—the 2-4.5 Shared Socioeconomic Pathway based on intermediate greenhouse gas emissions; (**E**,**F**)—the 3-7.0 Shared Socioeconomic Pathway based on high greenhouse gas emissions [76].

To predict some possible Italian locust distribution changes in 2021–2040 and 2041–2060, two climatic models (CNRM-ESM2-1 and GISS-E2-1-G) relative to several Shared Socioeconomic Pathways (1–2.6, 2–4.5, 3–7.0) were used. Their comparative analysis shows that the local parts of the range may shift northward and north-eastward (Figure 5 and Figure 6). However, according to the CNRM-ESM2-1 model (Figure 5), these shifts will be relatively weak (especially in comparison with the forecasted shifts of *Oedaleus decorus* distribution in West Siberia [80]), but according to the GISS-E2-1-G model (Figure 6), they will be rather significant. In any case, the main areas with suitable conditions for *C. italicus* can remain the same, but the level of their applicability may decrease slightly relative to the current situation.

As may be expected, the forecasted shifts grow significantly if the level of greenhouse emissions rises (from the 1–2.6 to 3–7.0 Pathways). They may also increase from now until the middle of 21st century. Almost all possible shifts are anticipated for West and Central Siberia. The local parts of the species range will be able to relocate northward and north-eastward to the modern forest-steppes and south taiga. As a result (especially after 2041 and if greenhouse gas emission will remain high), the Italian locust will able to penetrate into several new regions, namely the northern parts of the Tyumen Region, the southern parts of Tomsk, Kemerovo, Krasnoyarsk Regions, the Republic of Khakassia, and the north-western parts of the Irkutsk Region. Moreover, some areas where the species will be able to occur are in the lower part of the Amur River basin, in the southern part of the Russian Far East, and in the Ordos Plateau. However, all these areas are very far from the real eastern boundaries of the species range.

## 4. Discussion

Examination of published and unpublished data concerning the latitudinal, longitudinal, and altitudinal distribution patterns of the Italian locust in the easternmost part of its range for the last hundred years shows that there are no evident shifts of its range boundaries associated with global warming or this tendency is weak. The main changes look very limited and may be explained by local transformations of ecosystems that resulted from increasing human activity, for instance, agriculture field abandoning, overgrazing, and the development of irrigation systems. However, in the region, key alterations can be traced for terrains where the species outbreaks may start. In the end of the 20th century, these areas moved northward and northeastward, and, in addition, the severity of upsurges significantly increased [3,4]. The similar trend, especially intensifying the severity of eruptions, was revealed by M. V. Stolyarov [17] for the southern parts of European Russia. This contrasts with the noticeable changes in the distribution, especially altitudinal, of other locusts (*Locusta migratoria* (Linnaeus) and *Dociostaurus maroccanus* (Thunberg)) in the region [81]. However, ecological modeling based on climatic models associated with relatively high greenhouse gas emissions predicts the opportunity of possible northward and northeastward shifts of the Italian locust range in West and Central Siberia.

Previously, some shifts of the Italian locust range were forecasted for Russia and adjacent regions [82,83]. The authors tried to associate the range boundaries with climatic parameters, such as effective temperatures and precipitation levels during warm seasons. Their predictions were based on some assumption that the so-called climatic range of the species could increase in 1996–2015. However, the real data on the Italian locust distribution exhibit no explicit alteration in the species distribution from the first half of the 20th century until now, at least, in the eastern part of its range (see Section 3.1). In any case, the authors forecasted possible shifts of the range northward and northeastward for 2034–2053 and 2080–2099 (according to the 2-4.5 Shared Socioeconomic Pathway). These predictions are partly similar to the results of our modeling, showing some opportunity of relatively wide distribution of *C. italicus* over the southern part of central Siberia in the middle of the 21st century (cf. Section 3.3).

Liao Chunhua and co-authors [84] analyzed the so-called breeding suitability index distribution over Xinjiang (NW China) and showed that the areas with possible suitable conditions for the Italian locust are mainly distributed across piedmont plains of local mountain systems and occupy relatively wide territories than our models show, especially in the southern part of Xinjiang (Tarim Basin, or Altishahr).

Recently I. Klein and co-authors [85] tried to consolidate several different approaches to foretell suitable breeding terrains for the Italian locust in the Pavlodar Region (NE Kazakhstan). They analyzed the data on young hoppers’ distribution, estimated the so-called Habitat Suitability Index (HSI) [85] using the very wide set of environment variables including soil moisture, soil types, seasonal Net Primary Productivity, etc., and compared HSI with the actual land surface conditions derived from the Sentinel-2 data. The authors demonstrated that such a combined approach allows revealing fine details of the breeding area distribution. However, the general pattern of the Italian locust distribution uncovered for the region coincides with our results (cf. Figure 2).

The data for 2022–2023 concerning the altitudinal distribution of *C. italicus* in the mountains of the Ili River Basin (East Tien Shan in NW China) [86] generally confirm our results, but show that the species may be dominant not only at low altitudes, but also at an altitude of more than 1800 m.

Ecologo-geographic modeling allows for estimating the importance of some bioclimatic variables for possible predictions of long-term dynamics of the species populations. Several very ordinary parameters were and are commonly used to model population dynamics, e.g., annual mean temperatures and annual/seasonal precipitation amounts or solar activity characteristics [16,17]. Our results show the most important variables for all datasets compared (before 1961; 1961–1997; 1998–2022; all data until 2022) (Table 3 and Figure 4), which are annual mean temperatures (bio_1), mean diurnal range of temperatures (bio_2), and precipitation seasonality (bio_15). The first and last parameters are well known and widely used but the mean diurnal range of temperatures is not very ordinary, and neither are the amounts of precipitation during the driest months (bio_14), the driest quarter (bio_17), and during the warmest quarter, which are also significant. In the region, these parameters are evidently associated with each other because July and August, as a rule, are the driest and warmest months. In addition, for the dataset before 1961, the maximum temperatures of the warmest month (bio_5), temperature annual ranges (bio_7), and mean temperatures of the warmest quarter (bio_10) are important. In those years, the significance of such variables could reflect relatively low air temperatures [87], limiting the Italian locust development. Two latter sets (1961–1997 and 1998–2022) are characterized by minimum temperatures of the coldest month. This parameter is essential for the egg overwintering. All these variables may be useful for generating models of long-term dynamics of populations.

*C. italicus* was and is one of the most prominent pests in the region [1,2,3,4,7,9,29,88,89,90]. Long-term dynamic patterns of the spatial and temporal populations’ distribution should be considered across a range of scales from the global to the local one [2,27,91]. However, up to date, we cannot set applicable criteria where, when, why, and how to study, monitor, and control its long-term dynamics [2,3,4]. There are significant differences between both local populations and their regional systems in long-term and short-term dynamics [3,4,15]. Moreover, only a few datasets of long-term monitoring may be used to reveal local population changes. This is why opportunities of long-term prediction are significantly limited. That means that to forecast the Italian locust activity, it is necessary to organize some kind of a long-term monitoring system in different parts of the species range, at least for selected local populations, with provision for varieties of both natural and transformed ecosystems [2,3,86,92,93].

Furthermore, it should be noted that ecologo-geographic models should be generated separately for each species. When a set of species is used (e.g., [94,95]), especially if species are quite different relative to their bionomics (development strategies, food and habitat preferences, behavior, etc.), the results of modeling may be unacceptable and inapplicable for plant protection systems.

On the contrary, spatial forecasts may use data on species distribution (presence, presence/absence, or abundance) over its whole range or other areas. As a rule, such datasets are applicable, especially for widely distributed and/or abundant forms. They can serve as an extremely important component for predicting locust-related risks [91] and allow us to reveal areas where species populations may be numerous and abundant and where upsurges can start in favorable seasons [2,3,27]. In this case, additional sets of environmental data (solar radiation, altitudes, soil types, soil salinity, soil humidity, vegetation types, landscapes, especially transformed, etc.) (cf. [78,80,85]) may be useful for generating high quality models. However, to forecast possible shifts of species distribution for the middle or the end of the 21st century, the so-called future climate data based on different climatic models may be used, but these data are commonly limited by the standard set of climatic variables. As a result, we should use the same types of data for all periods or try to generate some models, considering possible changes in soils or vegetation types or other similar variables in the future. Furthermore, to predict the actual possible species distribution, the integration of ecologo-geographic modeling and opportunities of remote sensing [85,96,97,98] should be very effective as well. Such a complex approach in conjunction with opportunities of modern GISs can be very useful to reveal some general patterns of distribution of both abundant and rare species those often occur in the same habitats [99,100].

## Figures and Tables

**Table 1:** Main differences between *Calliptamus italicus italicus* and *C. abbreviatus*.

Species (Subspecies)	Size, mm [4,44]	Tegminal Length	Tegminal Form	Hind Wing Coloration
*C. italicus italicus* *	Bigger; body length: 14.5–28.7 (males); 23.5–41.6 (females); hind femora length: 12.1–15.0 (males); 13.8–24.6 (females)	Well developed, commonly with apices surpassing the knees of the posterior femora	Almost parallel-sided, their apices are usually broadly rounded	Usually pink
*C. abbreviatus*	Smaller; body length: 12.3–21.1 (males); 23.5–35.1 (females); hind femora length: 4.6–12.1 (males); 7.3–19.4 (females)	Relatively short, commonly with apices never surpassing the knees of the posterior femora	Narrowing to relatively acute apices	Usually colourless

* *C. italicus reductus* Ramme is distributed over the southern mountains of Central Asia (Pamiro-Alay, some parts of the Pamirs, N Afghanistan). This subspecies is smaller than *C. italicus italicus* and is characterized by reduced tegmina with apices never surpassing the knees of the posterior femora and narrowing to evidently acute apices.

**Table 2:** Upper altitudinal limits of the Italian locust distribution in the easternmost part of its range.

Mountain System	Region	Ridge	Year(s)	Northern Latitude	Upper Limit, m
Altai (Altay)	North-West	Kolyvanskij	1999–2000	51°20′	600
	South	Kurchumskij	1976	48°33′	1100
Tien Shan (Tian Shan)	North	Dzhungarian (Jungarian) Alatau (N)	1975	45°45′	~1300/ 1600–~2200
		Dzhungarian (Jungarian) Alatau (NW) [59]	1963–1964	45°31′	1500/ 1800–~2200
	East	Borohoro [60]	1981	44°27′	1500
		Eren Habirga Shan [78]	2013–2014	43°27′	1770
	North	Ketmen [61]	1948	43°15′	~2000
		Kirgiz (Kyrgyz) Alatau	1986	42°40′	1880
	West	Ferganskij	1986	41°18′	2200
Pamiro–Alay	Alay	Alay	1986	39°55′	2450
Pamirs	North	Darvaz	1991	38°40′	2900

**Table 3:** Predictive contributions of variables for three periods (before 1961, 1961–1997, 1998–2021) and for all data.

Variable	Variable Explanation	Distribution Before 1961		Distribution 1961–1997		Distribution 1998–2022		All Data	
		Percent Contribution	Permutation Importance	Percent Contribution	Permutation Importance	Percent Contribution	Permutation Importance	Percent Contribution	Permutation Importance
bio_1	annual mean temperature	29	55.9	6.3	19.6	13.3	9.7	26.3	43.4
bio_2	mean diurnal range (mean of monthly (max temp—min temp))	21.8	6	23	0.1	21.9	25.4	25.3	14.1
bio_3	isothermality (bio2/bio7) (×100)	0.2	1.3	3.8	23.6	0.2	1.5	0.5	1.5
bio_4	temperature seasonality (standard deviation ×100)	0.2	0.4	0.9	21.8	0.4	0.1	0.8	2.1
bio_5	max temperature of warmest month	0.5	4.8	0.1	0.5	0.1	0	0.1	0.8
bio_6	min temperature of coldest month	0.4	6	17	0.2	13.8	5.2	4.2	1.4
bio_7	temperature annual range (bio5-bio6)	7.3	1	0.5	0.3	1	0.3	0.9	0.1
bio_8	mean temperature of wettest quarter	2.6	0.7	0.1	0.4	0.3	0.9	0.1	0.1
bio_9	mean temperature of driest quarter	0.4	0	0.9	1.6	1.7	5.3	0.3	1
bio_10	mean temperature of warmest quarter	0.1	0.2	0	0	0	0	0	0
bio_11	mean temperature of coldest quarter	0	0	1.1	3.5	0.2	2.3	0.1	0
bio_12	annual precipitation	0.1	0	0.2	1.9	1.2	5.1	0.6	3.5
bio_13	precipitation of wettest month	0.3	1.2	0.2	0.1	1.6	1.3	0.6	0.1
bio_14	precipitation of driest month	7.5	1.7	0.3	0.7	7.6	0.6	3.8	0
bio_15	precipitation seasonality (coefficient of variation)	25.1	16.2	10.2	3.7	12.5	8.9	18.4	27.5
bio_16	precipitation of wettest quarter	0.3	0	4.8	8.7	4.4	11.7	0.1	0.1
bio_17	precipitation of driest quarter	0	0	22.3	1.3	8.7	4.2	10.9	0
bio_18	precipitation of warmest quarter	4	4.2	7.8	9.3	10.1	14.5	6.7	2.9
bio_19	precipitation of coldest quarter	0.5	0.1	0.8	0.3	4.1	12.6	3.3	14.8

In highlighted green are the five most significant variables for each group.

## Data Availability

Supplementary information or data can be provided upon request from the corresponding author.

## Supplementary Materials

The following supporting information can be downloaded at https://www.mdpi.com/article/10.3390/insects16020211/s1: Table S1: Geographic coordinates of known records of *Calliptamus italicus* in the easternmost parts of its range.
